# Twist and turn: a revised structural view on the unpaired bubble of class II CPD photolyase in complex with damaged DNA

**DOI:** 10.1107/S205225251800996X

**Published:** 2018-08-08

**Authors:** Manuel Maestre-Reyna, Junpei Yamamoto, Wei-Cheng Huang, Ming-Daw Tsai, Lars-Oliver Essen, Yoshitaka Bessho

**Affiliations:** a Institute of Biological Chemistry, Academia Sinica, 128, Academia Road, Sec. 2, Nankang, Taipei 115, Taiwan; b Division of Chemistry, Graduate School of Engineering Science, Osaka University, 1-3 Machikaneyama, Toyonaka, Osaka 560-8531, Japan; c Department of Chemistry, Philipps University Marburg, Hans-Meerwein Strasse 4, Marburg 35032, Germany; d LOEWE Center for Synthetic Microbiology, Philipps University Marburg, Hans-Meerwein Strasse 6, Marburg 35032, Germany

**Keywords:** class II CPD photolyases, DNA repair, DNA distortion, cyclobutane pyrimidine dimer, crystal structure

## Abstract

Cyclobutane pyrimidine dimer (CPD) photolyases harness the energy of blue light to repair UV-induced DNA CPDs. Upon binding, CPD photolyases cause the photodamage to flip out of the duplex DNA and into the catalytic site of the enzyme. This process, called base-flipping, induces a kink in the DNA, as well as an unpaired bubble, which are stabilized by a network of protein–nucleic acid interactions. Previously, several co-crystal structures have been reported in which the binding mode of CPD photolyases has been studied in detail. However, in all cases the internucleoside linkage of the photodamage site was a chemically synthesized formacetal analogue and not the natural phosphodiester. Here, the first crystal structure and conformational analysis *via* molecular-dynamics simulations of a class II CPD photolyase in complex with photodamaged DNA that contains a natural cyclobutane pyrimidine dimer with an intra-lesion phosphodiester linkage are presented. It is concluded that a highly conserved bubble-intruding region (BIR) mediates stabilization of the open form of CPD DNA when complexed with class II CPD photolyases.

## Abstract 2

Co-crystallization with the natural, phosphodiester-linked substrate combined with exhaustive computational analysis reveals how photolyases stabilize and lock the DNA unpaired bubble resulting from cyclobutane pyrimidine dimer binding and subsequent flip-out.

## 1. Introduction   

DNA photolyases are ancient and ubiquitous flavin adenine dinucleotide (FAD)-containing enzymes (Essen & Klar, 2006 ▸) which harness the energy of blue light to repair UV-induced DNA lesions in a sequence-independent manner. Furthermore, they constitute the oldest and best conserved DNA-repair pathway in any biological system (Mei & Dvornyk, 2015 ▸). DNA photolyases have evolved to respond to the two major UV DNA photoproducts, namely 6–4 pyrimidine–pyrimidone dimers (6–4 photoproducts) and cyclobutane pyrimidine dimers (CPDs). Accordingly, DNA photolyases can be functionally divided into (6–4) and CPD photolyases (Lucas-Lledó & Lynch, 2009 ▸). CPD photolyases can be further phylo­genetically assigned as class I, class II, cry-DASH and, recently, class III (Scheerer *et al.*, 2015 ▸). The closely related cryptochromes act mainly as blue-light photoreceptors *via* light-dependent reduction of the oxidized chromophore (photoreduction; Geisselbrecht *et al.*, 2012 ▸). Interestingly, cryptochromes can function as magnetoreceptors by light-induced formation of magnetosensitive radical pairs (Ritz *et al.*, 2000 ▸).

Although DNA photolyases are phylogenetically quite diverse (Kiontke *et al.*, 2011 ▸), they all share a common two-domain topology. Elements of both domains are involved in DNA recognition and binding, while each domain bears a single, light-harvesting cofactor as a chromophore (Geisselbrecht *et al.*, 2012 ▸; Kiontke *et al.*, 2011 ▸; Mees *et al.*, 2004 ▸). Universally, the C-terminal domain of DNA photolyases contains a reduced FAD cofactor (FADH^−^) in a unique U-shaped conformation (Mees *et al.*, 2004 ▸), which acts in both light absorption and catalysis. The cofactor of the N-terminal domain, the so-called antenna chromophore, varies according to the phylogenetic relationship (Kiontke *et al.*, 2014 ▸). Antenna chromophores increase the absorption cross-section of the enzyme. Highly efficient light-driven DNA repair is achieved by Förster-like energy transfer from the antenna cofactor to the catalytic FADH^−^, which absorbs blue light comparably weakly (Essen & Klar, 2006 ▸). During CPD DNA repair by CPD photolyases, FADH^−^ absorbs a single blue photon, yielding excited FADH^−^ (FADH^−^*). The cofactor then injects one electron into the bound CPD lesion, resulting in a radical pair, namely oxidized radical FADH (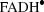) and the reduced radical anion lesion 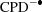 (Weber, 2005 ▸; Brettel & Byrdin, 2010 ▸). 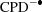 then splits almost barrierlessly into its constituent bases and the electron is back-transferred to the 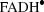.

All previously published CPD photolyase–DNA co-crystal structures show that in order to achieve the optimal FADH^−^ to CPD distance for electron transfer, both bases composing the CPD must enter the photolyase catalytic site by flipping out of the DNA strand at around a 120° angle (Kiontke *et al.*, 2011 ▸). As with other DNA-repair enzymes (Qi *et al.*, 2009 ▸), CPD photolyases appear to play an active role in unstacking the CPD, since the conformational space of photodamaged DNA does not allow spontaneous flipping of the CPD lesion out of the nucleotide strand (Knips & Zacharias, 2017 ▸). Additionally, it has been demonstrated that the inability to flip CPD out of dsDNA is a common feature of cry-DASH proteins, which act as photolyases that are solely specific for single-stranded DNA (Pokorny *et al.*, 2008 ▸). Interestingly, some ancestral cry-DASH proteins do retain dsDNA-repair activity (Tagua *et al.*, 2015 ▸), which further supports an active role in CPD flip-out for all other, dsDNA-repairing, CPD photolyases.

As a consequence of CPD photolyase binding and the subsequent base-flipping, the natural DNA distortion caused by the CPD is exacerbated. Free CPD-containing DNA has already been shown to present a kink, *i.e.* a sharp bend in the DNA backbone involving adjacent base-pair unstacking (Dickerson, 1998 ▸), of around 30° at the photodamage (Husain *et al.*, 1988 ▸; Park *et al.*, 2002 ▸). Photolyase-bound CPD DNA is further distorted, with a kink angle at the CPD lesion of roughly 50° (Kiontke *et al.*, 2011 ▸; Mees *et al.*, 2004 ▸). Furthermore, in class II CPD photolyases the DNA downstream of the CPD is dislocated, *i.e.* laterally bent, in comparison with class I CPD photolyases (Kiontke *et al.*, 2011 ▸). In both class I and class II CPD photolyases the empty space opened along the DNA strand owing to the unpairing and flipping of the CPD lesion, *i.e.* the unpaired bubble, is then stabilized by side chains residing within the loop connecting helices α17 and α18 (Kiontke *et al.*, 2011 ▸; Mees *et al.*, 2004 ▸). Class II CPD photolyases stabilize the bubble in the DNA strand *via* π-stacking of three conserved amino acids, Arg429, Trp431 and Arg441, within the previously mentioned loop (Supplementary Fig. S1*a*), as well as ionic interactions with the unpaired complementary bases (Kiontke *et al.*, 2011 ▸), whereas class I CPD photolyases use a different set of interactions (Essen & Klar, 2006 ▸).

These observations have been corroborated *via* the available high-resolution co-crystal structures of class I, class II and cry-DASH CPD photolyases in complex with DNA (Pokorny *et al.*, 2008 ▸; Kiontke *et al.*, 2011 ▸; Mees *et al.*, 2004 ▸; Selby & Sancar, 2006 ▸). However, mainly owing to the challenge in purifying chemically synthesized native CPDs, these complexes share the use of a synthetic CPD analogue in which the intra-lesion phosphodiester linkage has been substituted by an uncharged formacetal moiety. Thus, the negative charge of the DNA backbone at the CPD lesion site was eliminated. Nevertheless, and as mentioned above, base-flipping stabil­ization involves positively charged amino acids interacting with the unpaired bases in the immediate vicinity of the CPD backbone. Therefore, it is questionable whether the hitherto described CPD-binding mode and mechanism for stabilizing the single-stranded DNA at the catalytic site fully represent the physiological photolyase–DNA complex. In order to address this issue, we present here the first co-crystal structure of *Methanosarcina mazei* class II CPD photolyase (*Mm*CPDII) in complex with fully native, phosphodiester-containing CPD DNA at 2.7 Å resolution. As expected, the global conformation of the *Mm*CPDII–DNA complex resembles the previously published structure that harboured the formacetal linkage (Kiontke *et al.*, 2011 ▸). On one hand, our data shown here corroborate previous notions regarding the mode of binding of the *cis*–*syn*-cyclobutane adduct within the active site, including the presence and role of a unique six-water cluster (6WC). However, the binding site for the CPD lesion and its counterbases exhibits subtle differences in amino-acid side-chain and unpaired-base placement, as well as in the hydration sphere, which result in a more pronounced DNA kink. This led us to identify the loop connecting helices α17 and α18 as a highly conserved bubble-intruding region (BIR) that is responsible for unpaired bubble stabilization and phosphate recognition. Accordingly, the presence of the intra-lesion phosphodiester moiety within the CPD lesion profoundly affects the recognition of damaged DNA and is crucial for efficient DNA binding in class II CPD photolyases.

## 2. Materials and methods   

### 2.1. Synthesis and hybridization of photodamaged DNA   

DNA duplexes with the same sequence as that previously described for the structure with PDB code 2xrz (Kiontke *et al.*, 2011 ▸) were employed. The complementary, non-photodamaged DNA with the sequence d(5′-TGCGCGAAGCC­GAT-3′) was ordered in large quantities from Genomics Ltd, Taipei, Taiwan. On the other hand, the single-stranded, photodamaged DNA was synthesized in-house. The phosphoramidite building block for the *cis*–*syn* CPD was purchased from Glen Research, Sterling, Virginia, USA. Solutions of the CPD building block and nucleoside phosphoramidites (Glen Research) were installed on an Applied Biosystems 3400 DNA synthesizer and a 14-mer, d(5′-ATCGGCT<>TCGCGCA-3′), was synthesized according to the manufacturer’s instructions. After deprotection of the intra-phosphodiester group of the CPD moiety by treatment of the solid support with thiophenol, cleavage and deprotection were carried out. The oligonucleotide was analyzed and purified with a Gilson analytical HPLC system, on which a Waters μBondasphere C18, 5 µm, 300 Å column (3.9 × 150 mm) was installed. The column was run with a linear gradient of 5–13% acetonitrile in 0.1 *M* triethylammonium acetate pH 7.0 over 20 min. The pooled solution was dried by evaporation and the residue was passed through an NAP-10 (GE Healthcare, Buckinghamshire, England) and a cation-exchange (AG 50W-X2, Na^+^ form, Bio-Rad Laboratories, Hercules, California, USA) column. Both strands were solubilized in storage buffer (100 m*M* Tris–HCl pH 8.0, 100 m*M* NaCl), mixed in a 1:1 molar ratio and hybridized in a thermocycler by heating the solution to 95°C and then slowly decreasing the temperature to 25°C over a period of 3 h.

### 2.2. Protein production and purification   

Protein production and purification followed established guidelines (Kiontke *et al.*, 2011 ▸). Briefly, *Escherichia coli* BL21(DE3) cells were transformed with a pET-28-based construct containing the MM_0852 open reading frame, which codes for *Mm*CPDII (GenBank ID AAM30548.1). The protein was produced *via* autoinduction in TB medium at 25°C, with yields of above 100 mg protein per litre of culture. Cell pellets were resuspended in buffer (50 m*M* phosphate buffer pH 8.0, 300 m*M* NaCl) and lysed. After the removal of cell debris *via* a second centrifugation step, supernatants were loaded onto a self-packed 10 ml nickel–NTA column and the protein was eluted *via* the addition of 250 m*M* imidazole to the running buffer. As a final polishing step, the protein was loaded onto a size-exclusion chromatography column containing Superdex 200 equilibrated with 10 m*M* Tris–HCl pH 8.0, 100 m*M* NaCl.

### 2.3. Protein crystallization and data acquisition   

Protein–DNA complexes were prepared in the dark and under oxidizing conditions (*i.e.* exposed to air) in order to avoid spontaneous DNA repair by mixing the protein solution with a 1.25× molar excess of dsDNA in such a way that the final protein concentration was between 6.0 and 6.5 mg ml^−1^. After 30 min incubation in the dark, crystals were grown *via* vapour diffusion in 4 µl drops consisting of 2 µl protein–DNA sample solution and 2 µl crystallization buffer [0.1 *M* sodium acetate pH 4.6, 0.25 *M* ammonium sulfate, 4%(*w*/*v*) PEG 4000]. After 24 h, crystals were fished out with loops and cryoprotected in crystallization buffer supplemented with 30%(*v*/*v*) glycerol, and data were measured either on the TPS-05A beamline at NSRRC, Taiwan or on BL32XU at SPring-8, Japan.

### 2.4. Data processing and structure solution   

The data acquired from several crystals were processed manually *via* a slightly modified version of the *KAMO* merging protocol (Yamashita *et al.*, 2018 ▸) using *XDS* (Kabsch, 2010 ▸). *BLEND* clustering (Foadi *et al.*, 2013 ▸) from the *CCP*4 suite (Winn *et al.*, 2011 ▸) was used to determine the best combination of data sets, which were then merged *via* *XSCALE* (Kabsch, 2010 ▸). The final merged and scaled data set was then solved *via* molecular replacement using *Phaser* (McCoy *et al.*, 2007 ▸). The molecular model with two *Mm*CPDII–CPD DNA complexes per asymmetric symmetry unit was further improved using a mixture of manual refinement in *Coot* (Emsley *et al.*, 2010 ▸) and least-squares automated refinement with *REFMAC*5 in *CCP*4*i* (Murshudov *et al.*, 2011 ▸). Here, FAD was modelled in its fully oxidized form and is shown as such in all figures. Data-collection and refinement statistics are provided in Supplementary Table S1.

### 2.5. Molecular-dynamics simulations   

All molecular-dynamics simulations were performed with the *Amber*17 software (Case *et al.*, 2017 ▸) using the newest versions of the Amber force fields for proteins (ff14SB; Maier *et al.*, 2015 ▸) and nucleotides [ff14 with the ∊/ζ_OL1_ (Zgarbová *et al.*, 2013 ▸), χ_OL3_ (Zgarbová *et al.*, 2011 ▸), χ_OL4_ (Krepl *et al.*, 2012 ▸) and β_OL1_ (Zgarbová *et al.*, 2015 ▸) updates (Cheatham & Case, 2013 ▸)], with additional parameters for FADH^−^ and for cyclobutane pyrimidine dimers with phosphodiester and formacetal backbones (Miyazawa *et al.*, 2008 ▸). The latter were manually updated for compatibility with the *Amber*17 nomenclature and for the presence of either backbone moiety. The simulations were run on a workstation equipped with four Nvidia GTX1080 GPUs running Ubuntu 16.04 LTS.

#### 2.5.1. System setup   

The *Mm*CPDII–DNA complex with a phosphate backbone was set up by extracting complex I (corresponding to protein chain *A* and DNA chains *C* and *D*; Supplementary Fig. S2) from the coordinates file. Alternative conformations were manually erased and protonation states were adjusted according to the *PROPKA* server (Olsson *et al.*, 2011 ▸; Søndergaard *et al.*, 2011 ▸) predictions for pH 4.6, the same as the crystallization condition. The processed file was then loaded into the *Amber* topology and coordinate preparation program *xleap* (Wang *et al.*, 2006 ▸) along with the corresponding parameter files for the cofactors and CPD. The system was neutralized by adding counter-charges in the form of sodium and/or chloride ions to give an effective sodium chloride concentration of 30 m*M*. Finally, the complex was enclosed in a TIP3P water box which extended 10 Å from the *Mm*CPDII–DNA complex. The formacetal CPD derivative of the *Mm*CPDII–DNA complex was prepared analogously, with the only difference being the parameter set for the CPD monomer. For both cases, sets of topologies and initial coordinates were finally produced.

#### 2.5.2. Minimization   

The topologies and coordinates were minimized and equilibrated individually prior to each production run. Minimization was performed in four steps. Firstly, solute atoms were constrained with a 100 kcal mol^−1^ harmonic restraint while *SHAKE*-restrained (Miyamoto & Kollman, 1992 ▸) waters and ions were allowed to relax for 500 cycles of steepest descent followed by 4500 steps of conjugate gradient. Next, restraints on ions, waters and DNA were lifted and the molecules were allowed to relax under the same conditions as before. The same procedure, including the FAD residue, followed. Finally, harmonic restraints on the protein were lifted and the whole system was relaxed for the same number of cycles.

#### 2.5.3. Equilibration and production dynamics   

After minimization, the temperature was slowly raised over 50 ps from 0 to 300 K using a Langevin thermostat (with random seed and γ = 5 ps^−1^), applying weak restraints to the solute molecules. Next, another 50 ps of constant-volume simulation at 300 K was performed in order to further equilibrate the system. Finally, constant-pressure, restraint-free equilibration to one atmosphere was carried out for 50 ps (Monte Carlo barostat, pressure-relaxation time 2 ps).

Following temperature and pressure equilibration, a period of 20 ns was allowed for final convergence of the system. After this, 200 ns of production dynamics were collected, with snapshots every 100 ps. This process, including minimization, was repeated twice for the simulation describing the complex with the phosphodiester-linked CPD, as well as for the formacetal-linked CPD, resulting in 400 ns of production dynamics for each. Overall, 0.8 µs of simulation time was probed.

### 2.6. Data analysis   

Angle, vector and water-density analyses were performed *via* *CPPTRAJ* (Roe & Cheatham, 2013 ▸), which is part of the *Amber*16/*AmberTools*17 package (Case *et al.*, 2017 ▸). For kink and dislocation angles, the centres of mass (COMs) of the base pairs at the 3′ and 5′ ends of each of the arms were used to generate vectors from which angles were derived. The twist angle within the unpaired bubble was calculated as the vector product between vectors generated between the COM of the CPD intra-lesion phosphodiester (P0; PDB entry 5zcw) or formacetal (C0; PDB entry 2xrz) and the combined COM of dG6′, dA7′, dA8′ and dG9′ of the corresponding structure. All protein figures were rendered non-orthoscopically with *PyMOL* (Schrödinger), while molecular-dynamics trajectories were analyzed, and videos were rendered, with *VMD* (Humphrey *et al.*, 1996 ▸).

Water-density analysis was performed *via* *GIST* (Nguyen *et al.*, 2012 ▸) as provided in *CPPTRAJ*. Briefly, 2000 snapshots from each 200 ns production-dynamics simulation were extracted, centred to their centre of mass and aligned using a randomly chosen snapshot. By aligning and centring all snapshots to the same set of coordinates, we could accurately compare *GIST* results using identical computation regions. Overall, the water-density analysis area spanned a 20 × 10 × 20 Å box centred on the protein–DNA interface, with individual densities being calculated in 0.5 × 0.5 × 0.5 Å voxels. In order to visualize the results, normalized water-density two-dimensional plots were constructed out of the *xz* plane.

Cavity data were computed using the *POVME* 2.0 software (Durrant *et al.*, 2014 ▸). Here, the same snapshots as for the water-density analysis were used, by which we could accurately compare *POVME* results using the identical inclusion regions. Two different volumes were calculated: (i) the unpaired bubble volume, corresponding to the total cavity left by base flipping, and (ii) the free volume, corresponding to the solvent-accessible volume, *i.e.* that which was not occupied by the stabilizing residues Arg429, Trp431 and Arg441. For the latter the simulation snapshots were not modified, while for the former the three amino acids were modified *in silico* to alanines. The pocket was then defined by a set of two overlapping inclusion spheres with radii from 5 to 6 Å, which covered the volume encompassing the protein–DNA interface. *POVME* was run with all default settings, a voxel grid spacing of 1.0 Å and the ConvexHullExclusion option set to ‘true’.

## 3. Results   

### 3.1. Overall structure of the *Mm*CPDII–DNA complex with a phosphodiester-linked CPD lesion   

**Figure 1:**
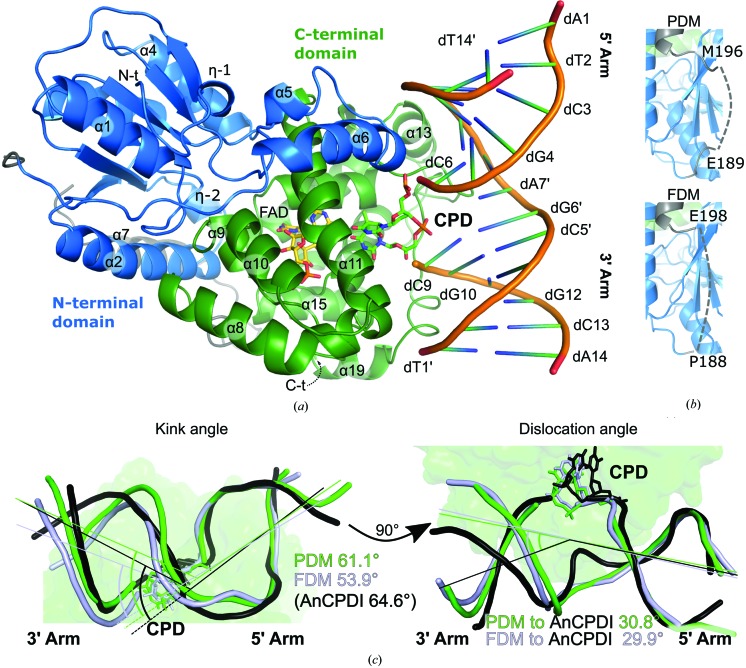
Overall fold of *Mm*CPDII in complex with double-stranded DNA containing a native cyclobutane pyrimidine dimer (CPD). (*a*) View of the overall complex, with the two subdomains in blue (N-terminal) and green (C-terminal). The DNA is shown as a double-stranded cartoon, while the oxidized FAD cofactor (yellow) and the CPD damage (green) are shown as stick models. (*b*) Differences in the domain-linker region (grey) in the PDM structure (PDB entry 5zcw) *versus* the FDM structure (PDB entry 2xrz). (*c*) DNA geometry distortions as a result of photolyase binding. Double strands are shown in green (PDM), pale blue (FDM) and black (*A. nidulans* class I CPD photolyase; *An*CPDI). For orientation purposes, the outline of the *Mm*CPDII structure is shown in pale green in the background. The side view (left) shows the kink angle, *i.e.* the sharp bend in the DNA resulting from CPD flipping and partial unstacking of the complementary adenines. Here, kink angles were calculated from the vector products of the 5′ *versus* 3′ arms of each chain. The top view (right) shows the dislocation of the 3′ arm in class II CPD photolyase (green, PDM; pale blue, FDM) when compared with class I (black, *An*CPDI). Dislocation angles were calculated by superposing the 5′ arms of all three molecules, followed by determining the vector products between either the PDM or FDM 3′ arm and the *An*CPDI 3′ arm.

The crystal structure of the *Mm*CPDII complex containing native CPD, *i.e.* presenting a phosphodiester linkage within the CPD moiety, here designated PDM (PDB entry 5zcw) was determined from crystals belonging to space group *P*2_1_2_1_2_1_, the same crystal form as that obtained for its formacetal linkage-containing equivalent (FDM; PDB entry 2xrz), but with limited isomorphism owing to changes in unit-cell parameters of up to 2 Å. Accordingly, both crystal structures comprise two *Mm*CPDII–CPD DNA complexes per asymmetric unit (Supplementary Fig. S2), with that corresponding to protein chain *A* showing better defined DNA termini (Fig. 1 ▸). For simplicity, from this point on the protein–DNA complex formed by protein chain *A* and DNA chains *C* and *D* will be called complex I, while that comprising protein chain *B* and DNA chains *E* and *F* is named complex II (Supplementary Fig. S2). Since the PDM electron density for complex II was less well defined than that for complex I, unless explicitly stated otherwise all descriptions presented here refer to complex I. Although the resolution of the PDM structure was worse than that of FDM (2.7 *versus* 2.2 Å), we were able to observe the entire double-stranded DNA (14-mer) in complex I, showing both the 5′ arm (DNA upstream of the CPD damage) and the 3′ arm (DNA downstream of the CPD damage; Fig. 1 ▸ *a*) to be fully defined by electron density. Meanwhile, PDM electron density for major parts of the 5′ arm was missing in complex II (Supplementary Fig. S2). Furthermore, in complex I we found significant electron density for a larger part of the linker region connecting the N- and C-terminal domains in chain *A* (amino acids Val186–Glu231; Supplementary Fig. S1*a*), with only amino acids between Glu189 and Met196 missing, while in FDM the residues between Pro188 and Glu198 are undefined (Fig. 1 ▸ *b*).

Despite the similarity between the two crystal structures, and the fact that there are no major inter-domain conformational changes when PDM is compared with FDM (r.m.s.d. of 0.73 Å for 856 common C^α^ atoms), there are obvious differences in the bound DNA geometry. In our calculations, the FDM kink angle derived from CPD binding was 53.9°. Meanwhile, PDM shows a markedly different value of 61.1° (Fig. 1 ▸ *c*, left). On the other hand, the DNA dislocation is very similar when compared with *Anacystis nidulans* class I CPD photolyase, with the dislocation in PDM corresponding to 30.8° and that in FDM to 29.9° (Fig. 1 ▸ *c*, right; Supplementary Fig. S1*a*). Since DNA kinking is a direct result of photolyase binding (Mees *et al.*, 2004 ▸; Kiontke *et al.*, 2011 ▸; Park *et al.*, 2002 ▸), we hypothesized that the observed differences must be related to the way that the CPD phosphodiester moiety (P0) is recognized at the active site *versus* the way that the formacetal-linked CPD can be accommodated in the same position.

### 3.2. Binding mode of the phosphodiester-linked CPD at the *Mm*CPDII binding site   

**Figure 2:**
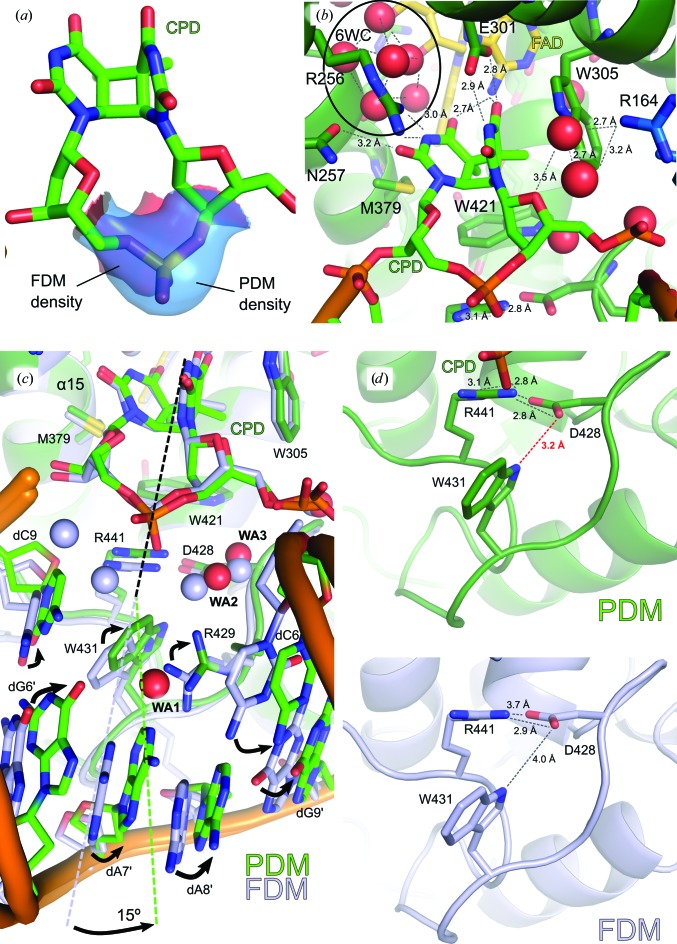
Natural CPD binding by *Mm*CPDII. (*a*) Determining the nature of the CPD backbone. Electron density for PDM (blue; PDB entry 5zcw) was much more prominent than for FDM (red; PDB entry 2xrz), clearly confirming the presence of a phosphodiester backbone. Electron densities were calculated as OMIT maps at a 1.5σ contour level against their respective models and then superposed with the PDM structure. (*b*) The PDM active site. The active site of the co-crystal structure contained the CPD (bright green) as well as an oxidized FAD cofactor (gold). It also included the class II CPD photolyase-specific water cluster (6WC). (*c*) Stabilizing the distorted DNA in the presence of a native CPD. Superposition of the active-site residues of PDM (green) and FDM (pale blue) reveals an ∼15° twist in the DNA geometry, which is accompanied by side-chain and base rearrangements (black arrows). Furthermore, the immediate hydration sphere also shifts, facilitating new interactions (WA1, WA2 and WA3 in red for PDM and in pale blue for FDM). (*d*) The lock bolt of the BIR. Top, the BIR lock bolt in the presence of a phosphodiester-linked CPD lesion (PDB entry 5zcw). The differential locking interaction between Asp428 and Trp431 is highlighted in red. Bottom, the BIR lock bolt in the presence of a formacetal-linked CPD lesion (PDB entry 2xrz).

Close examination of the PDM structure shows an increase in electron density at the backbone between the two thymines comprising the CPD in each complex, clearly indicating the presence of the full phospho­diester linkage at this position (Fig. 2 ▸ *a*, Supplementary Fig. S3). When comparing PDM complexes I and II, the binding mode is very similar (Fig. 2 ▸ *b*; r.m.s.d. of 0.296 Å for 408 common C^α^ atoms). Here, much like in FDM, the CPD occupies a cavity within 12.1 Å of the centre of mass of the FAD cofactor, with the C4 carbonyl groups of both the 5′ and 3′ CPD thymines interacting with the N6 amino group of the adenine of FAD (Fig. 2 ▸ *b*). Prominent protein–CPD lesion interactions include π-stacking (Trp421 from behind and Trp305 from the side), hydrophobic interactions (Met379), hydrogen bonds (Asn257 and Glu301) and ionic interactions (Arg164 and Arg256), all of which play a role in keeping the CPD moiety within the active site. The active-site six-water cluster (6WC) is also present in the PDM structure (Fig. 2 ▸ *b*). The 6WC participates in the binding of the 3′ CPD thymine, but has also been proposed to act as a proton donor during the DNA-photorepair catalytic cycle (Kiontke *et al.*, 2011 ▸). Much like in the FDM structure, the CPD damage appears to be intact. In accordance with the low conformational selectivity of *Mm*CPDII for CPD, PDM presents a quasi-canonical CPD photodamage, with a tilt of 50.6° between the base planes (FDM tilt 43.1°; the CPD tilt within small-molecule crystal structures is ∼57°; Kiontke *et al.*, 2011 ▸; Park *et al.*, 2002 ▸). Given the almost identical binding modes in either PDM or FDM, the binding mode of the CPD moiety within the active site is almost unaffected by the nature of the other *Mm*CPDII–DNA backbone interactions.

In contrast, the crucial stabilization of the unpaired bubble that results from CPD flipping into the photolyase active site and from the corresponding unpaired adenines on the complementary DNA strand is strongly affected by the chemical nature of the intra-lesion linkage, *i.e.* the phosphodiester moiety (P0) *versus* the formacetal group (C0) (Fig. 2 ▸ *c*). Most of these changes take place in the loop connecting helices α17 and α18, *i.e.* the bubble-intruding region (BIR), which is directly in contact with the unpaired bubble (Fig. 2 ▸ *c*, Supplementary Fig. S1). Previously, two alternative conformations had been observed for BIR-mediated stabilization of the flipped CPD and unpaired bubble in complexes I and II of FDM (Kiontke *et al.*, 2011 ▸). In PDM, however, we observe a third conformation for BIR in both complexes, which is similar, but not identical, to that in FDM complex II (Fig. 2 ▸ *c*). In order to compare these two modes, the active sites of PDM complex I and FDM complex II were aligned, including the CPD, the FAD and protein side chains with atoms within a 3.5 Å radius of either of the above, but not within the space left by CPD flipping (Fig. 2 ▸ *c* and Supplementary Fig. S3). Relative to the CPD backbone, the guanidine moiety of PDM Arg441 is 1 Å closer to the P0 centre of mass, allowing the amino acid to form a salt bridge with it. Furthermore, the PDM Arg429 side chain is shifted by 1.6 Å. Simultaneously, the Arg429 side chain can now directly interact with N1 in the unpaired dA7′ and also indirectly with dA7′ N6 *via* a single crystallographic water (WA1; Fig. 2 ▸ *c*) and with the intra-lesion phosphodiester *via* a pair of water molecules (WA2 and WA3; Fig. 2 ▸ *c*). Further, the Arg429 shift causes a rearrangement of the base immediately upstream of the CPD, dC6, which shifts away from the unpaired bubble by 1.8 Å. Conversely, the Trp431 side chain, which interacts with the CPD downstream base dC9, is pulled by Arg429, causing it to also shift by 1.8 Å into the space left by CPD flipping and allowing it to interact with the carboxylic side chain of Asp428 *via* its indole N^∊^ atom (Fig. 2 ▸ *d*). Set into the spatial relationship to the active site, these subtle rearrangements result in the entire complex being twisted by ∼15° around the PDM active site (Fig. 2 ▸ *c*). As a consequence, the unpaired space volume, *i.e.* the volume resulting from the unpaired DNA bubble, shrinks from 802 Å^3^ in FDM to 772 Å^3^ in PDM (Table 1 ▸), leading to the differences in DNA-binding mode and ultimately to the difference in DNA geometry and distortion.

### 3.3. Dependence of *Mm*CPDII protein breathing on the nature of the intra-CPD linkage   

As a next step to probe *Mm*CPDII–DNA interactions in a less constrained environment, we performed four 200 ns rounds of production molecular-dynamics (MD) simulations based on PDM (Supplementary Videos S1–S4). Two of the rounds presented PDM without any further modifications (PDM simulations), while in the other two rounds the CPD phosphodiester link in PDM was substituted by a formacetal moiety, resulting in an FDM equivalent system (FDM′).

**Figure 3:**
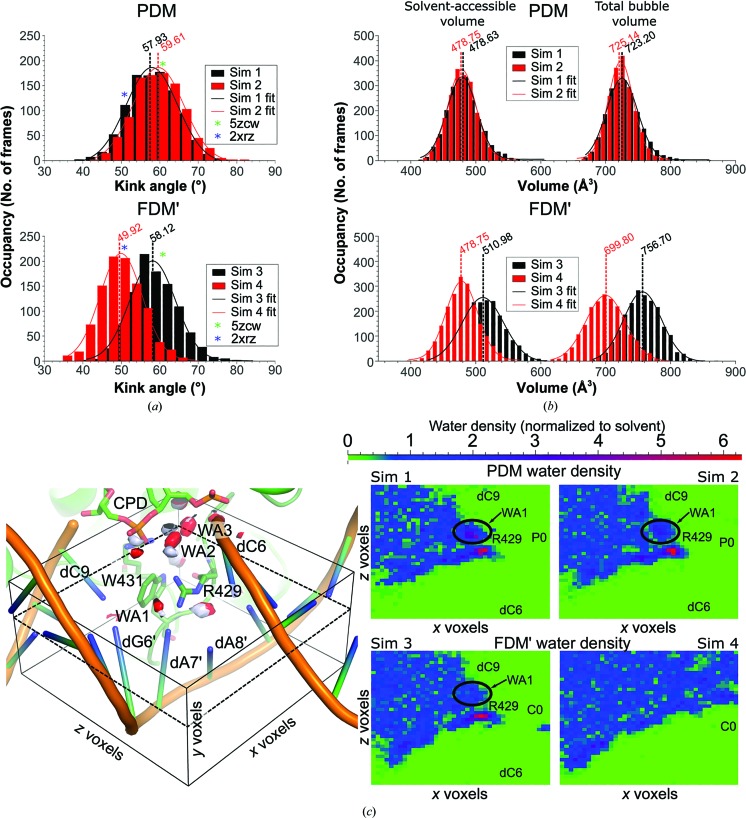
Simulating the breathing behaviour of CPD phosphodiester or formacetal linkage-containing dsDNA–*Mm*CPDII complexes. (*a*) DNA kink-angle population analysis, with the middle of the distribution highlighted by a dotted line and value in the corresponding colour. The bins corresponding to either the PDM (PDB entry 5zcw, complex I) or FDM (PDB entry 2xrz, complex II) crystal structures are highlighted with green and blue asterisks, respectively. Top, populations for CPD phosphodiester-containing simulations (simulation 1 in black, simulation 2 in red). Bottom, populations for two of the CPD formacetal-containing simulations (simulation 3 in black, simulation 4 in red). (*b*) Non-occluded and total volume population analysis for the unpaired DNA bubble. Non-occluded volumes (*i.e.* the volume not covered by the amino acids occluding the unpaired space) are always smaller and on the left side of the graph. The total volume is always larger and on the right side of the graph. The middle point of the distribution is highlighted *via* a dotted line and the corresponding value. Top, populations for the two phosphodiester-containing simulations (simulations 1 and 2 in black and red, respectively). Bottom, populations for two of the formacetal-containing simulations (simulations 3 and 4 in black and red, respectively). (*c*) Water-density analysis for the phosphodiester- and formacetal-containing systems. Left, overall representation of the water-density analysis system. An analysis box (black lines) was drawn roughly around the area of interest, comprising the unpaired bubble and portions of the surrounding solvent. The box was divided into voxels of 0.5 × 0.5 × 0.5 Å. Here, red objects show regions of the analysis box containing waters with an occupancy higher than 5 in simulation 1 (containing phosphodiester) and pale blue objects those in simulation 3 (containing formacetal). Right, highest water occupancy *y*-axis slice at the 12th *y* voxel (dotted plane on the left panel), shown for each of the simulations. High-occupancy voxels are shown in red and voxels with solvent-equivalent densities are shown in blue. Voxels occupied by solute, *i.e.* with water densities below 1, are shown in green. For orientation purposes, the approximate positions of dC6, dC9, C0, P0 and Arg429 are shown on the map, although they are not necessarily coplanar with the 12th *y* voxel.

Here, the PDM system behaved very similarly to the experimentally determined crystal structure, maintaining all of its key features (Fig. 3 ▸, Supplementary Videos S1 and S2). Firstly, and most noticeably, population analysis of the DNA kink angle showed that it obeyed a centrosymmetric normal distribution with an average centre at 58.77 ± 0.30° (Table 1 ▸, Fig. 3 ▸ *a*). By the same process, we determined the average unpaired volume space to be 724.17 ± 0.81 Å^3^, with BIR residues Arg429, Trp431 and Arg441 occupying a total volume of 245.48 ± 1.12 Å^3^ and leaving 478.69 ± 0.77 Å^3^ solvent-accessible (Table 1 ▸, Fig. 3 ▸ *b*). Furthermore, water-density analysis showed that all three crystallographic high-occupancy waters (WA1, WA2 and WA3 in Fig. 2 ▸ *c*) were also present (Fig. 3 ▸ *c*). The position occupied by the solvent-exposed WA1, which bridges one of two interactions between dA7′ and Arg429, had a water oxygen density that was ∼6.5 times higher than that of bulk solvent water (6.5 × SW).

In order to further understand the differences between PDM and FDM, we next modified the PDM initial topology and coordinates by substituting the CPD phosphodiester P0 for a formacetal group (C0), effectively transforming the PDM CPD into the FDM CPD while leaving both the PDM DNA geometry and the hydration sphere intact (FDM′ simulations). We then proceeded to perform 400 ns of production MD for this system *via* two 200 ns replicates and to follow any perturbations resulting from the exchange of –PO_2_– to –CH_2_– (Supplementary Videos S3 and S4). Interestingly, we observed two remarkably different behaviours. In the first of the two 200 ns trajectories (Supplementary Video S3), the DNA kink angle was not noticeably different from PDM (58.12 ± 0.26°, Fig. 3 ▸ *a*). However, the positions of WA2 and WA3 had shifted by ∼15°, as in PDB entry 2xrz, while WA1, although in a similar position, presented a much lower occupancy (∼3.5 × SW; Fig. 3 ▸ *c*). Finally, the overall unpaired bubble volume had increased to 756.70 ± 0.59 Å^3^ (Table 1 ▸, Fig. 3 ▸ *b*), which a Student’s t-test revealed to be a significant change when compared with the PDM simulation. Nevertheless, the space occluded by Arg429, Trp431 and Arg441 within the unpaired bubble remained constant when compared with the PDM trajectories, at 245.72 ± 0.91 Å^3^, indicating that a larger fraction of the bubble remained solvent-accessible (510.98 ± 0.69 Å^3^; Table 1 ▸).

Early on in the second 200 ns FDM′ trajectory (Supplementary Video S4) the dC9–dG6′ pairing was broken, with dC9 proceeding to form an anomalous pairing with dA7′ (Supplementary Fig. S4*a*). As a result, the shape and volume of the unpaired bubble shrank (699.80 ± 0.50 Å^3^; Table 1 ▸, Fig. 3 ▸ *c*), which led to a more acute DNA kink angle (49.92 ± 0.17°; Fig. 3 ▸ *a*). At the same time, however, BIR side chains Arg429, Trp431 and Arg441 occluded less of the unpaired bubble (221.05 ± 0.74 Å^3^), indicating that they had receded from the unpaired space. Concomitantly, the presence of high-occupancy waters was profoundly diminished, and while WA2 and WA3 remained in their established positions, the occupancy of WA1 was barely above that of bulk solvent (Fig. 3 ▸ *c*). The absence of WA1, along with the radically different shape of the unpaired region, resulted in Arg429 interacting almost exclusively with dA8′ and not with dA7′ (Supplementary Video S4).

Overall, therefore, the presence of a formacetal group at the CPD backbone profoundly affected the simulations, perturbing the DNA geometries and the stabilization of the unpaired bubble and affecting its hydration sphere.

## 4. Discussion   

The function of CPD photolyases is well known (Zhong, 2015 ▸; Brettel & Byrdin, 2010 ▸), in particular owing to the availability of high-resolution co-crystal structures in which CPD-containing DNA is bound to the active site of CPD photolyase (Kiontke *et al.*, 2011 ▸; Mees *et al.*, 2004 ▸). However, a significant drawback of these structures is that in all cases the CPD DNA used in the co-crystals lacks the naturally occurring form of the CPD lesion, instead comprising a chemically synthesized analogue in which the negatively charged intra-lesion phosphodiester linkage between the bases has been replaced by a neutral formacetal moiety. How this lack of the natural backbone affects DNA binding and recognition remained unclear, although it was presumed to exert a minor effect. Our first structure of a CPD photolyase, *Mm*CPDII, in complex with a native phosphodiester-linked CPD shows there are considerable differences in how the unpaired DNA bubble is stabilized by class II photolyases.

Since the pyrimidine-dimer moiety is identical in natural CPD and its artificial counterpart, it is hardly surprising that the *Mm*CPDII active site is capable of accommodating both equally well (Fig. 2 ▸). However, the obvious differences in volume, hydrophobicity and charge between the formacetal and phosphodiester groups in the DNA backbone (Fig. 2 ▸ *a*) result in considerable structural changes in the unpaired DNA bubble which appears after CPD base-flipping (Fig. 2 ▸ *c*).

The presence of three amino acids, Arg429, Trp431 and Arg441, is key in plugging the unpaired bubble by interacting with both neighbouring bases (Arg429 with either dA7′ or dA8′ and Trp431 with dC9) and the flipped-out backbone, *i.e.* phosphodiester in natural CPD or the formacetal linkage in the synthetic CPD analogue (Arg441; Fig. 2 ▸). Overall, these amino acids are responsible for occluding around one third of the bubble volume (Table 1 ▸, Fig. 3 ▸), while the remainder is solvent-accessible. Further, their conformation, along with that of the flipped-out CPD, is responsible for the characteristic kink angle of DNA bound to photolyase (Kiontke *et al.*, 2011 ▸).

Stabilizing DNA distortions is also an essential feature of many other DNA-binding proteins (Werner *et al.*, 1996 ▸; Rohs *et al.*, 2009 ▸), and has been ascribed to either residues with the capacity for π-stacking or those with positive charges (Luscombe *et al.*, 2001 ▸; Werner *et al.*, 1996 ▸). However, the role of protein–DNA interface hydration in distortion stabilization has recently come into the spotlight (Schwabe, 1997 ▸; Schneider *et al.*, 2014 ▸; Jayaram & Jain, 2004 ▸; Chong & Ham, 2016 ▸). High-occupancy waters have been found in diverse roles, such as lubrication (Schwabe, 1997 ▸), adapters (Halford & Marko, 2004 ▸) or the occlusion of otherwise empty spaces left by DNA distortion (Robinson *et al.*, 1998 ▸; Chen *et al.*, 2005 ▸). It is therefore of interest that when comparing both the crystal structures and the MD simulations of PDM and FDM′, the presence of the formacetal moiety on the CPD yields lower occupancies for key stabilizing waters, especially WA1 (Fig. 3 ▸ *c*). In the context of photolyase activity, the high occupancy of WA1 is quite remarkable, as photolyases must act on four possible substrates, *i.e.* TT and the less common CT, TC and CC pyrimidine dimers, and therefore must contend with stabilizing considerably different unpaired bubbles (AA, AG, GA and GG, respectively). Here, WA1 may act as a highly versatile adapter between Arg429 and the 5′ region of the unpaired bubble. For example, in the presence of TC CPD damage Arg429 would need to interact with a 5′dG in the GA unpaired bubble. Under these conditions, the Arg429–WA1–5′G interaction could be easily adapted by a slight shift in position between the three partners, providing full stabilization of dG N1, N2 and O6 (Supplementary Fig. S4*b*). Thus, this highly conserved, Arg429–WA1-based class II mechanism may indicate a higher tolerance for different CPD substrates than in class I CPD photolyases, where substrate specificity is high for TT dimers and stabilization of the unpaired bubble appears to be a fairly unspecific and passive affair (Essen & Klar, 2006 ▸; Kim & Sancar, 1991 ▸).

Additionally, when we perturbed the PDM structure by substituting the phosphodiester by a formacetal linkage in our simulations, we observed that the complex was incapable of maintaining WA1 in its assigned position (Fig. 3 ▸ *c*). Furthermore, the simulation appeared to react to the absence of these waters in two distinct ways. It either maintained the PDM kink angles, at the cost of a large, unstable, solvent-accessible volume in the unpaired bubble (Fig. 3 ▸ *c*, Table 1 ▸), as in simulation 3 (Supplementary Video S3), or it modified the overall geometry of the complex, approaching FDM kink angles (Table 1 ▸, Supplementary Video S4). In the latter case the change in DNA geometry resulted in Arg429 receding towards dA8′, causing Trp431 to follow (Fig. 3 ▸, Supplementary Fig. S4). An unstable, wobbling, dC9 then entered the unpaired bubble, interacting anomalously with dA7′. The resulting unpaired bubble is smaller than in the phosphodiester CPD-containing simulations (Table 1 ▸). Interestingly enough, Arg429, Trp431 and Arg441 occlude a smaller volume, but the inclusion of dC9 fully compensates for this. Thus, our simulations suggest that in the presence of the formacetal backbone the *Mm*CPDII–DNA complex is faced with a seemingly impossible task: it may either maintain the physiological DNA distortion (kink angle) at the expense of a fully occluded unpaired space or the opposite, but not both.

Although it is difficult to demonstrate that dC9 wobbling behaviour could occur *in vitro*, it is perhaps owing to these changes that in the FDM crystal structure, but not in the PDM crystal structure, two different conformations are observed between complex I and complex II (Supplementary Fig. S5). Previously, these two conformations had been hypothesized to correspond to a closed (complex II) and a bolted (complex I) state (Kiontke *et al.*, 2011 ▸), with the second being the truly active state. Crucially, in the bolted conformation dC9 in FDM is not fully paired owing to a lateral shift with respect to its complementary dG6′ (Supplementary Fig. S5), while Watson–Crick pairing is fully realized in both PDM complexes and in the FDM bound complex (complex II). We suggest, therefore, that the bolted conformation, with its Trp431 rotated by 90°, does not represent the fully active protein but is instead a conformation which solves the problem of bubble occlusion *versus* kink angle in the presence of a formacetal CPD backbone. As such, this conformation is not representative of how *Mm*CPDII binds its natural substrate, but rather is an example of enzyme plasticity when faced with an unusual substrate.

So far, the specific role of particular side chains in the stabilization of the unpaired bubble in photolyase–DNA complexes has not yet been appropriately addressed (Essen & Klar, 2006 ▸). However, extensive analysis of all class II CPD photolyase sequences shows that the BIR, to which Arg429, Trp431 and Arg441 of *Mm*CPDII belong, exerts a remarkable conservation pattern that is consistent with the mode of unpaired bubble stabilization as described above (Supplementary Fig. S1*b*). Of the two arginines, only Arg441, which forms the directed salt bridge to the intra-lesion phosphodiester backbone, is strictly conserved. The other, Arg429, can be replaced by other space-demanding residues such as glutamine, methionine or histidine. Furthermore, the majority of class II photolyases mostly harbour the residue corresponding to Trp431 followed by the other aromatics phenyl­alanine and histidine. Interestingly, only the PDM structure shows the Trp431 N^∊1^ atom of the indole moiety forming a hydrogen bond to Asp428 (Fig. 2 ▸ *d*), which, although not directly entering into the unpaired bubble, is part of the BIR. Asp428 is strictly conserved in all class II photolyases and forms a salt bridge with Arg441 (Fig. 2 ▸ *d* and Supplementary Fig. S1*b*). From this, we can infer that the triad of Asp428, Trp431 and Arg441 forms a bolt-like substructure which aids in stabilization of the unpaired bubble. Although Arg429 is replaceable, at least in class II photolyases, by other voluminous residues, their function of interacting with the unpaired bases is still crucial in maintaining unpaired bubble stability. In class I photolyases an alternative conservation pattern for their BIR has emerged (Supplementary Fig. S1*b*), as exemplified by the *An*CPDI–DNA complex. Interestingly, in this structure harbouring the intra-lesion formacetal linkage, one of the conserved BIR residues, Arg404, is the only residue that lacks any interaction with DNA by being packed to the protein surface. Clearly, this residue could fulfil the same function as Arg441 in *Mm*CPDII in stabilizing the intra-lesion phosphodiester by adopting an alternative side-chain conformation.

In conclusion, by combining structural and computational studies, we have presented here an examination of the ways in which class II CPD photolyases bind their native substrate. Furthermore, we have shown that while previously published structures were accurate in their description of class II CPD photolyase active sites, this was not the case for the entirety of the binding area. Finally, we have demonstrated that *Mm*CPDII, a nonspecific, double-stranded DNA-binding protein, uses tools that agree with the overall view of how this class of proteins affect DNA ultrastructure. By allowing us to identify the hitherto unnoticed BIR, these results will pave the way for further refinement or rethinking of the structural biology of the binding of photodamaged DNA by CPD photolyase in terms of the roles of peripheral effectors and water in the protein–DNA complex.

## 5. Related literature   

The following references are cited in the Supporting Information for this article: Crooks *et al.* (2004 ▸) and Essen *et al.* (2017 ▸).

## Supplementary Material

PDB reference: *Methanosarcina mazei* class II CPD photolyase in complex with intact phosphate-containing CPD lesion, 5zcw

Supplementary Table and Figures.. DOI: 10.1107/S205225251800996X/mf5026sup1.pdf

Supplementary Video S1.. DOI: 10.1107/S205225251800996X/mf5026sup2.mp4

Supplementary Video S2.. DOI: 10.1107/S205225251800996X/mf5026sup3.mp4

Supplementary Video S3.. DOI: 10.1107/S205225251800996X/mf5026sup4.mp4

Supplementary Video S4.. DOI: 10.1107/S205225251800996X/mf5026sup5.mp4

## Figures and Tables

**Table 1:** Geometric parameters for the *Mm*CPDII–DNA binding site Kink angle refers to the angle between the downstream and upstream arms of the bound DNA. Volumes were calculated as the total volume of the cavity resulting from CPD flip-out (unpaired bubble) or the volume not occluded by Arg429, Trp431 and Arg441 (free volume). Calculations for simulations are based on population analysis, as shown in Fig. 3 ▸.

System	Kink angle (°)	Unpaired bubble (Å^3^)	Free volume (Å^3^)
PDM	61.1	772	
FDM	53.9	802	
Simulation 1 (phosphodiester)	57.93 ± 0.20	725.14 ± 0.56	478.75 ± 0.60
Simulation 2 (phosphodiester)	59.61 ± 0.220	723.20 ± 0.59	478.63 ± 0.48
Average of simulations 1 and 2	58.77 ± 0.30	724.17 ± 0.81	478.69 ± 0.77
Simulation 3 (formacetal)	58.12 ± 0.26	756.70 ± 0.59	510.98 ± 0.69
Simulation 4 (formacetal)	49.92 ± 0.17	699.80 ± 0.50	478.75 ± 0.55
