# Supplementary material for: Twist and turn: a revised structural view on the unpaired bubble of class II CPD photolyase in complex with damaged DNA
Source: IUCrJ. 2018 Aug 8;5(Pt 5):608–18. doi: 10.1107/S205225251800996X (PMC6126647; doi:10.1107/S205225251800996X)
Supplement: Supplementary file 1 [file m-05-00608-sup1.pdf]

IUCrJ

Volume 5 (2018)

Supporting information for article:

Twist and turn: a revised structural view on the unpaired bubble of class II CPD-photolyase in complex with damaged DNA

Manuel Maestre-Reyna, Junpei Yamamoto, Wei-Cheng Huang, Ming-Daw Tsai, Lars-Oliver Essen and Yoshitaka Bessho

Table S1 Data collection and refinement statistics for the *Mm*CPDII-DNA complex (5ZCW)

High resolution statistics are shown in parenthesis.

Data collection and processing statistics	
Wavelength (Å)	1.000
Resolution (Å)	44.57 - 2.7 (2.796 - 2.700)
Space group	P 21 21 21
Unit cell (Å)	71.1 114.42 166.42 90 90 90
Total number of reflections	260676 (13981)
Unique reflections	38064 (3734)
Multiplicity	6.8 (3.7)
Completeness (%)	99.89 (99.87)
Mean I/σ	10.67 (1.43)
Wilson B-factor	44.82
R-merge	0.5437 (0.9290)
R-meas	0.5883 (1.085)
R-pim	0.2219 (0.5563)
CC1/2	0.857 (0.522)
CC*	0.961 (0.828)
Refinement statistics	
Reflections used in refinement	38051 (3732)
Reflections used for R-free	1882 (184)
R-work	0.2191 (0.3271)
R-free	0.2555 (0.3363)
CC(work)	0.870 (0.547)
CC(free)	0.886 (0.524)
Number of non-hydrogen atoms	8323
Macromolecule atoms	7785
Ligand atoms	195
Solvent atoms	343
Protein residues	888
RMS(bonds)	0.013
RMS(angles)	1.52
Clashscore	2.62
Number of TLS-groups	6
Ramachandran statistics (%)	
Favored	97.95
Allowed	2.05
Outliers	0.00
Rotamer outliers	2.7
B-factors	
Average	75.22
Macromolecule	76.35
Ligand	64.94
Solvent	55.27

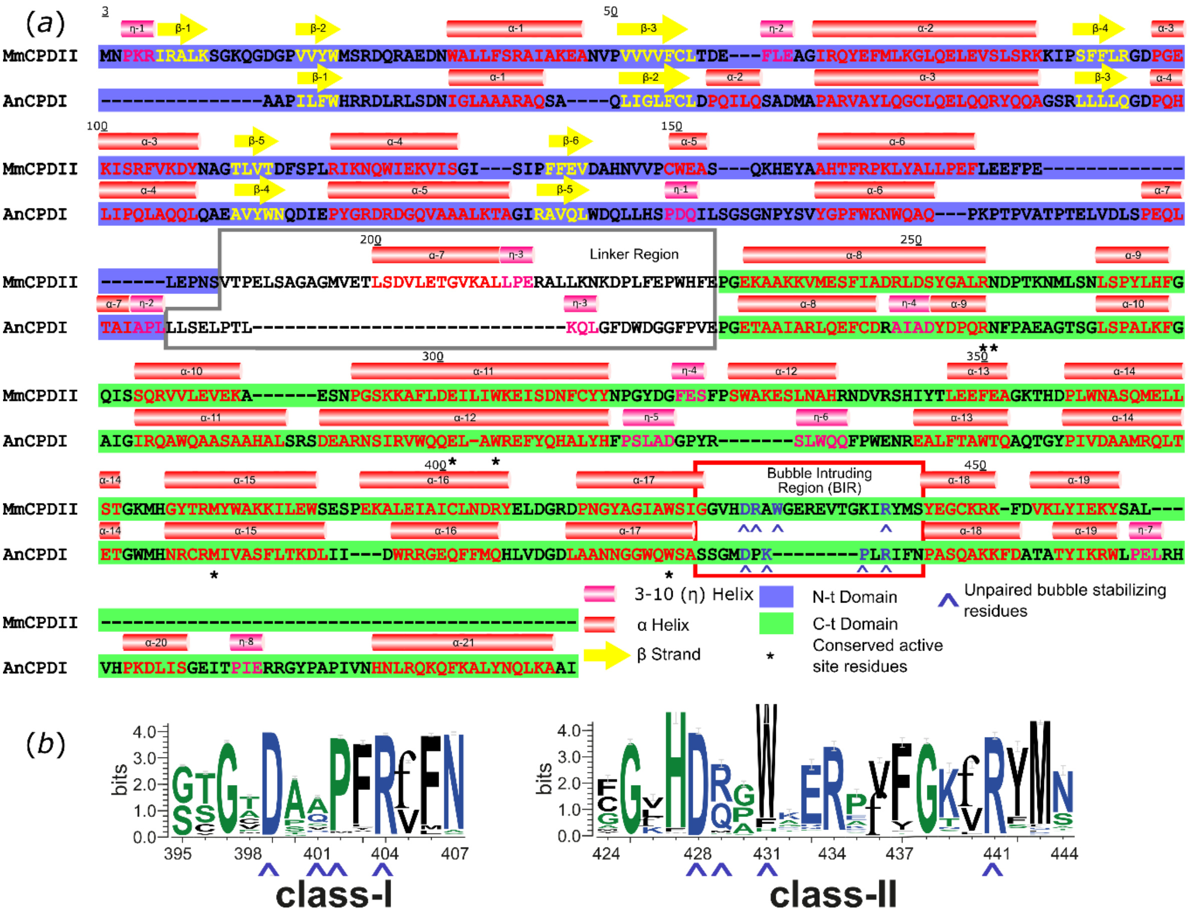

Figure S1 Sequence analysis of CPD-photolyases. (*a*) Structural alignment of *Mm*CPDII against the *Anacystis nidulans* class I CPD-photolyase (*An*CPDI). The alignment was performed via a local copy of T-Coffee, using its Expresso variant52. For both proteins, the same loop is used for stabilizing the DNA unpaired bubble (red square), however, the actual residues used during the stabilization, as highlighted in light blue within the box, are not conserved between classes. The linker between the N- and C-terminal domains is highlighted by the gray box. (*b*) Sequence logos for the bubble intruding regions of class I and II CPD-photolyases. The height at each position corresponds to the abundance of the corresponding residue in different class I and II orthologs. 3349 sequences of class I and 451 sequences of class II CPD-photolyases were isolated from a sequence-similarity network generated on the whole photolyase-cryptochrome superfamily (Essen *et al.*, 2017); the pairwise sequence identity of these non-redundant sequences is less than 90%. Multiple-sequence alignments as generated by Clustal Omega were used in WebLogo 3 (Crooks *et al.*, 2004) using the *Mm*CPDII and *An*CPDI sequences as references. Below each of the logos, blue arrows highlight the same amino-acids as in A. Letter coloring follows the default WebLogo 3 hydrophobicity-dependent scheme, with hydrophilic amino-acids in blue, neutral in green, and hydrophobic in black.

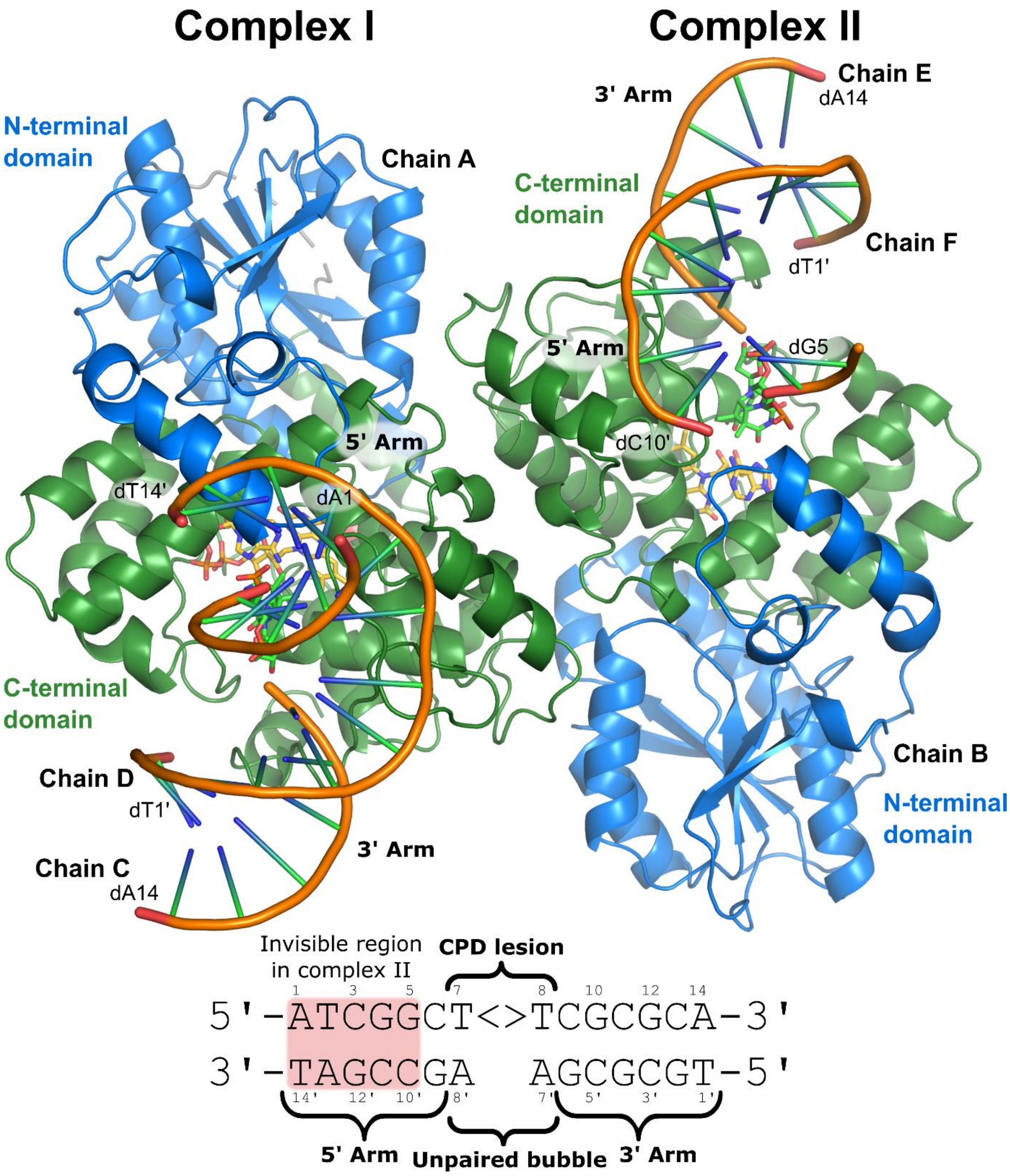

Figure S2 *Mm*CPDII-DNA complex crystal features. Top: PDM A.S.U. showing the complex I on the left, and complex II on the right. In both, the color scheme follows Fig. 1, with the N-terminal and C-terminal domains blue and green, respectively. As in Fig. 1, the CPD lesion is highlighted as a green licorice model, while the FAD cofactor as a gold one. Bottom: Sequence and details of the CPD-lesion containing DNA fragment. Regions mentioned in the text and other figures are marked in brackets, and the poorly defined region in complex II as a pale red square.

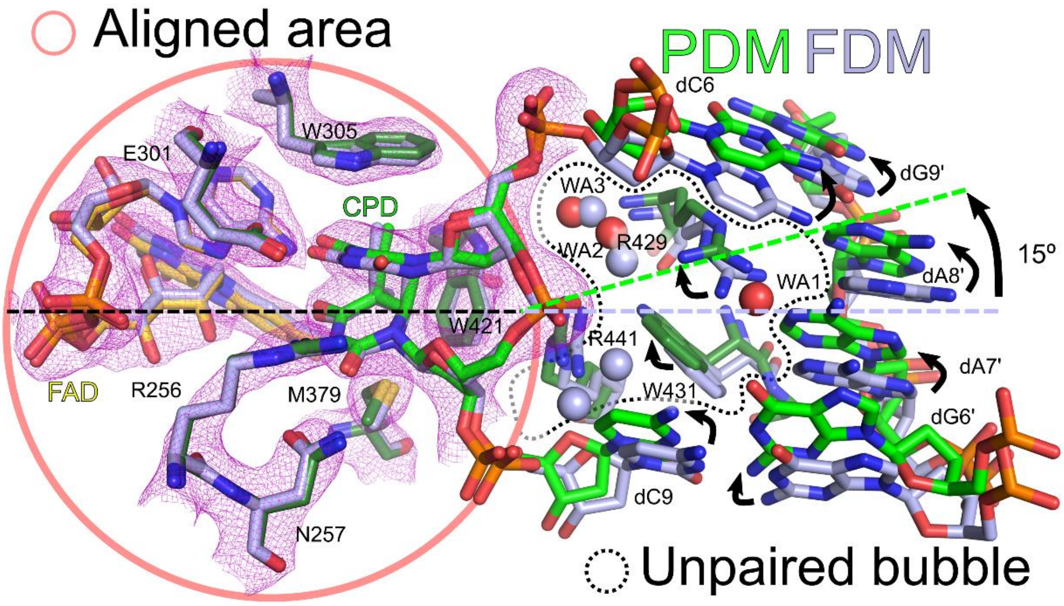

Figure S3 Alignment between PDM and FDM. In order to observe the subtle changes in the stabilization of the DNA unpaired bubble (dotted line) in the PDM and FDM complexes, the active site (red circle) of both complexes are aligned to each other. As can be observed from the SIGMAA-weighted 2mF_{obs}-DF_{calc} map of the PDM active site (purple mesh), both active sites fit perfectly within the same electron density.

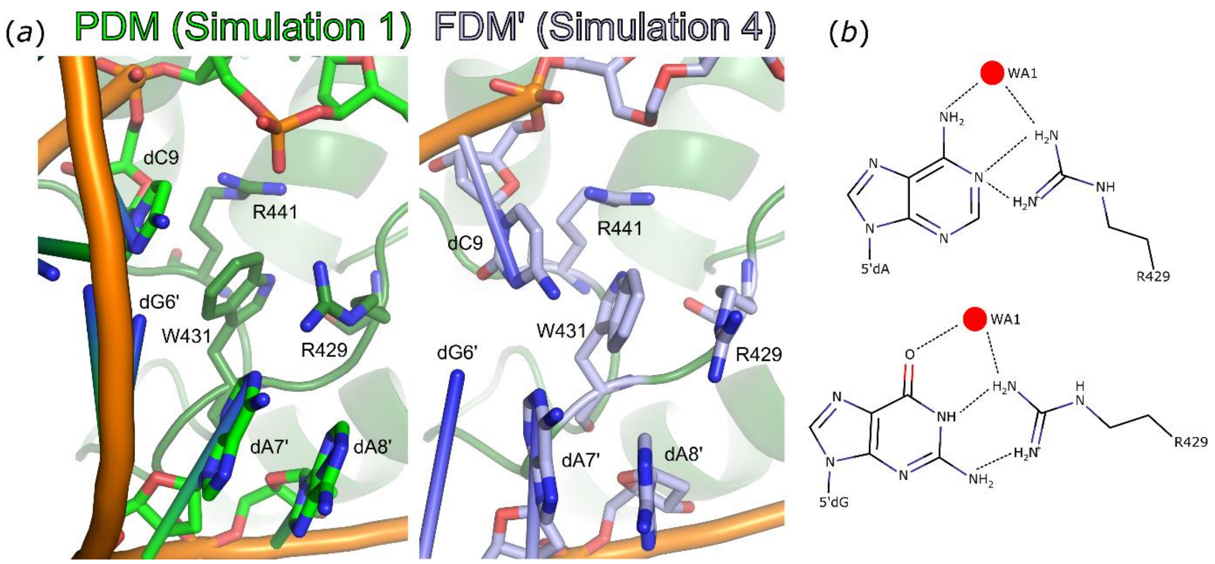

Figure S4 Plasticity of the unpaired bubble. (*a*) Stabilization of the DNA unpaired bubble during *Mm*CPD-DNA simulations. Left: In the presence of the native, phosphodiester containing CPD, the unpaired bubble is stable, and Watson-Crick pairing between dC9 and dG6' is maintained. Right: In the presence of an artificial CPD, the unpaired bubble becomes unstable. One of the ways in which it was stabilized in the simulation was by dC9 interacting with the unpaired dA7'. (*b*) Possible alternative stabilization modes by R429-WA1 for a 5'A (top) or 5'G (bottom) in the unpaired bubble.

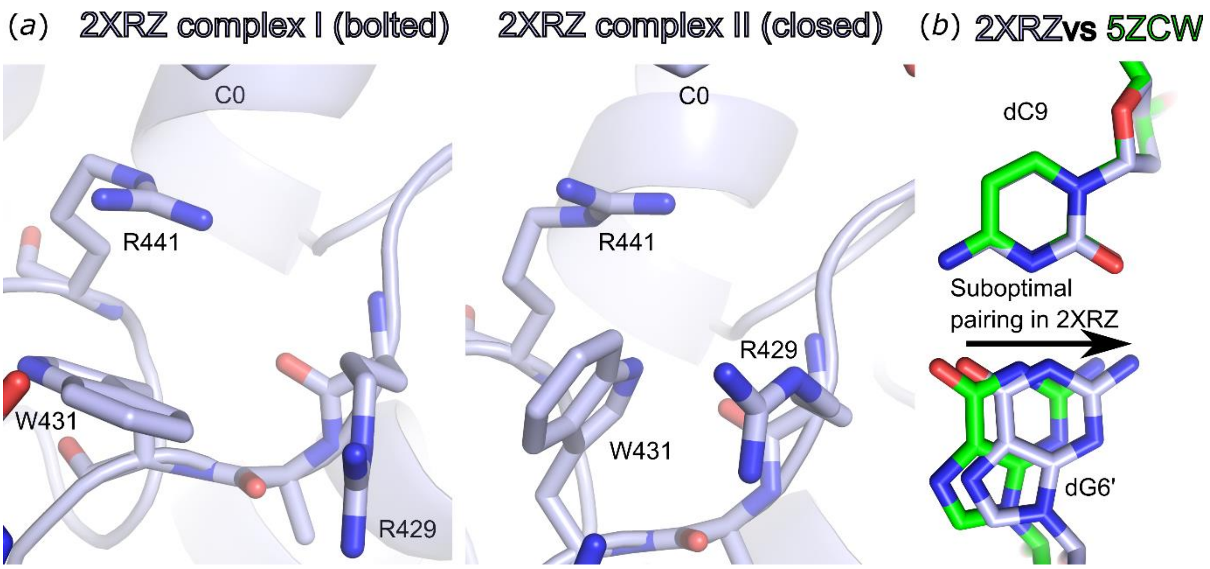

Figure S5 Features of the previously reported 2XRZ structure. (*a*) In the 2XRZ structure (equivalent to FDM), the two complexes in the asymmetric unit showed two different conformations in the unpaired bubble. Previously, it had been hypothesized that the bolted conformation in complex I was the more physiological one, while the closed one in complex II an intermediate stage in the process of DNA binding. (*b*) In the 2XRZ complex I (pale blue) base pairing between the CPD adjacent base pair dC9-dG6' is suboptimal due to sliding. On the other hand, base pairing on the same position for 5ZCW (green) is fully canonical.
